# “If I Didn′t Do Sports, I Would Definitely Eat Less Meat”—Physical Activity: Enemy or Ally for Healthier Food Choices?

**DOI:** 10.3390/nu17213362

**Published:** 2025-10-25

**Authors:** Maria Elide Vanutelli, Roberta Adorni, Viviana Carolina Cambieri, Marco D’Addario, Patrizia Steca

**Affiliations:** Department of Psychology, University of Milano-Bicocca, 20126 Milan, Italy; maria.vanutelli@unimib.it (M.E.V.); viviana.cambieri@unimib.it (V.C.C.); marco.daddario@unimib.it (M.D.); patrizia.steca@unimib.it (P.S.)

**Keywords:** meat consumption, plant-based proteins, masculinity, omnivores, veg*ans, beliefs, focus group

## Abstract

**Background:** Plant-based diets have been widely recommended as sustainable, ethical, and health-conscious alternatives. However, their adoption remains limited due to nutritional and cultural concerns, which may be reinforced in contexts associated with masculinity. This study aimed to explore (a) whether physical activity represents a barrier to plant-based food choices, and (b) whether beliefs about plant-based diets differ between sedentary and physically active individuals. **Methods:** A mixed-methods study was conducted, combining focus groups and survey data. Twenty-six Italian volunteers were divided into four groups: (I) physically active omnivores; (II) sedentary omnivores; (III) physically active veg*ans; and (IV) sedentary veg*ans. The discussions focused on protein-rich foods (animal-based vs. plant-based) and addressed nutritional concerns related to athletic performance, as well as attitudes toward plant-based proteins and veg*an individuals. **Results:** Omnivores—particularly active ones—favored animal-based proteins, perceiving them as more palatable, nutritious, and better suited for physical performance, a view often reinforced by coaches. This group also emphasized sensory pleasure as a barrier to adopting plant-based alternatives. It addressed masculinity-related themes more frequently; moreover, while veg*an participants were more inclined to seek professional advice to ensure a balanced diet, omnivores referred to the traditional Mediterranean diet as their primary source of nutritional guidance. This factor may further hinder openness to plant-based choices. **Conclusions:** The findings highlight stronger stigma among physically active individuals and suggest the need for interventions that replace the current narrative of plant-based diets as a restrictive or dietetic alternative with one that frames it as compatible with strength, vitality, and overall well-being.

## 1. Introduction

### 1.1. Plant-Based Diets: Opportunities and Resistances

Global population growth and current patterns of food consumption are exerting increasing pressure on natural resources, biodiversity, and agricultural systems [1,2]. Intensive livestock production, in particular, accounts for about 31% of total anthropogenic greenhouse gas emissions [3] and 57% of food-related emissions [4]. Additionally, it utilizes nearly 80% of the global agricultural land for grazing or feed production [5]. In addition to its environmental consequences, serious ethical concerns must be addressed regarding the treatment of animals, which are subjected to severely compromised living conditions throughout their short lifespans [6,7]. For these reasons, international recommendations encourage a transition toward more plant-based diets [8], rich in legumes, whole grains, fruits, and vegetables, which are associated not only to reduced environmental footprint [9], but also numerous health benefits such as lower risks of type 2 diabetes [10], cancer [11], and cardiometabolic disease [12,13]. Additionally, previous research has estimated that transitioning from a typical omnivorous to a plant-based diet could result in a 53% reduction in projected greenhouse gas emissions [14,15].

However, despite these documented advantages, the global population continues to struggle with implementing these recommendations in daily life. In Italy, for example, only 7.2% of the population identifies as vegetarian, and 2.3% as vegan [16]. This resistance cannot be explained solely by a lack of information. Indeed, social and psychological factors also contribute to shaping various forms of resistance directed both at vegan individuals and at plant-based foods themselves.

Previous research has documented the presence of negative biases against veg*an individuals, who are often evaluated as negatively as, or even more negatively than, other minority groups [17]. The stigma is higher among people endorsing conservative or right-wing ideologies, who tend to perceive them as a symbolic threat to traditional social norms [17]. Among the Italian veg*an population, 76.4% report having experienced some form of intolerance, with varying frequency and severity [16].

These stereotypes extend beyond people to food. Meat-eaters frequently perceive plant-based products as less tasty or satisfying, particularly when their identity or values are challenged [15]. This negative expectation, together with the perception of plant-based diets as restrictive, may contribute to framing dietary change as a form of renunciation rather than a positive transformation.

### 1.2. Masculinity, Meat Consumption, and the Role of Sports

This dynamic becomes particularly evident when considering gender-related constructs. Historically, meat has carried strong symbolic meanings of power, status, social dominance [18,19,20], and masculinity [15,18,21]. Several studies have shown that men consume higher amounts of meat [22] and associate red meat more strongly with their gender identity [23,24]. Moreover, greater self-perceived masculinity predicts stronger resistance to reducing meat consumption, a pattern that intensifies when masculinity is perceived as threatened, leading to compensatory increases in meat choice [25,26]. Furthermore, prior research has documented more negative attitudes toward veganism among men [20,27,28,29], as well as more negative evaluations of vegan men compared to vegan women [17].

The norms surrounding masculinity are particularly salient in sports settings. Sport has historically embodied values of competitiveness, physical toughness, aggression, and an emphasis on winning, traits aligned with hypermasculine ideologies [23,25]. These values often translate into the widespread belief that animal-based proteins are essential for muscle development and optimal performance.

From a nutritional standpoint, physical activity increases protein requirements [30] and athletes may require up to twice the protein intake of sedentary individuals [31,32]. A prevailing view in the sports context holds that animal-based proteins are superior, and that an omnivorous diet is essential for achieving athletic goals [33,34]. Conversely, plant-based diets are often considered nutritionally inferior [35,36], despite growing evidence supporting their adequacy for athletic performance when planned adequately in terms of energy needs, variety, and supplementation [33,37,38,39], especially with respect to vitamin B12. Thus, the link between physical activity and meat consumption appears to be supported by cultural and symbolic factors, as well as practical and nutritional considerations, often reinforced by professionals such as coaches, team physicians, and nutritionists [33].

Although the associations between meat consumption and masculinity, as well as between sport and masculinity, have been extensively examined, the direct link between physical activity and attitudes towards plant-based diets still needs further investigation. A recent qualitative study [33] has begun to address this issue by interviewing professional athletes following plant-based, omnivorous, or mixed diets. Participants consistently endorsed the view that protein is essential for athletic performance yet differed in their acceptance of plant-based sources. Notably, many equated proteins with animal products, revealing a conceptual overlap between the two. The topic of masculinity was rarely mentioned and explicitly endorsed in only one instance.

However, in this study, all participants were professional athletes, which means they belonged to a specific group of individuals. Naive perceptions of the relationship between plant-based diets and physical activity among members of the general population who engage in physical activity and recreational sports may be more common and, therefore, equally relevant. Moreover, the sample primarily included participants from Northern Europe, where dietary habits are strongly characterized by animal-protein consumption; different patterns might therefore emerge in regions with distinct cultural and dietary traditions.

### 1.3. The Current Study

Building on the literature reviewed above, the present study was guided by two main research questions: (I) Does physical activity represent a barrier to plant-based choice? (II) What are other barriers to plant-based choice? How do they vary by lifestyle?

To answer these research questions, we conducted a qualitative study involving four focus groups composed of participants who differed in both dietary regimen (omnivorous vs. vegetarian/vegan, hereafter veg*an) and lifestyle (active vs. sedentary). Focus group methodology is particularly well-suited to capturing complex and multidimensional constructs. Within a group dynamic, not only are explicit beliefs expressed, but latent motivations often surface, primarily through the interactive exchange of opinions [40].

The present study contributes to the existing literature by including not only athletes but also individuals who engage in regular physical activity, as well as sedentary participants, in the sample. Indeed, cultural representations may also influence individuals who are not directly engaged in physical activity. Moreover, the empirical investigation focuses on the Italian context, which is characterized by a strong and deeply rooted culinary tradition [41]. This cultural background may, on the one hand, facilitate openness toward plant-based choices—given the naturally high presence of plant-based dishes in the Mediterranean diet—but, on the other hand, could serve as a parallel reference model that further reinforces attachment to traditional eating patterns and hinders openness to dietary change. Furthermore, the discussion was deliberately focused on protein-rich food sources, comparing animal-based and plant-based proteins [42]. This emphasis allowed us to frame the conversation around the substitution of key macronutrients, rather than general food preferences, thereby addressing the specific nutritional concerns frequently cited in discussions of athletic performance and plant-based diets. Finally, we adopted a mixed-methods approach. In addition to the qualitative discussion, participants completed a brief quantitative survey during the focus group sessions, designed to assess explicit attitudes and common stereotypes regarding plant-based protein and veg*an individuals. The survey items also served as prompts to stimulate discussion on themes that might not have emerged spontaneously otherwise.

Given that prior research has identified cultural identification with meat-eating as a significant barrier to adopting more sustainable diets [43], we predicted that participation in physical activity might further reinforce meat consumption as a normative behavior. Consequently, we expected that for physically active individuals, the perceived loss associated with giving up meat would be greater, due to both symbolic and nutritional considerations, and the theme of masculinity would be more frequently discussed.

## 2. Materials and Methods

### 2.1. Participants

Twenty-six Italian volunteers, currently residing in Northern Italy (10 men, 15 women, and one non-binary person; Mage = 29.69, SD = 5.29), participated in the study (Table 1). They were assigned to one of four groups based on their dietary regimen and physical activity levels. The four groups were composed as follows: (I) physically active omnivores; (II) sedentary omnivores; (III) physically active veg*ans; (IV) sedentary veg*ans. To create balanced groups, sampling was performed by also considering age and gender.

The inclusion criterion for assessing physical activity was to participate in sports training at least twice a week. The inclusion criterion for assessing the diet group was self-identification as an omnivore or veg*an. Age (being 18 to 45 years old) represented a further inclusion criterion for all groups.

### 2.2. Procedure

The study was approved by the local commission for minimal-risk studies of the Psychology Department of the University of Milano-Bicocca and consisted of the following steps:

(I). Recruitment and informed consent

Participants were recruited by snowball sampling. Before the meetings, participants were required to read and sign the informed consent form. Then, they were asked to fill in demographic information (gender and age). Both the informed consent and the questionnaire were delivered via the Qualtrics platform. Once each group was complete (minimum sample size = 6; men/women ratio ≥ 0.5), participants received a link to fill out a group poll, where they could indicate their preference for the date and time of the focus group. After setting the appointment, participants received a reminder a few days prior, along with a new link to access the meeting.

(II). Focus Group

By following the link received via email, participants were encouraged to access the meeting a few minutes earlier to check for audio and video quality. The focus groups were conducted online via Cisco WebEx (Version 43.11) and lasted about one hour. The interviews started in November 2023 and ended in April 2024. They were audio-recorded through an offline digital device, and the recording was deleted after being transcribed and integrated with notes.

Participants were required to be equipped with two devices: a computer or tablet to access the WebEx meeting, and a cell phone to interact with the group. In fact, at specific times during the interview, they were asked to frame QR codes to access Mentimeter. This platform provides tools to engage the audience during meetings or conferences. This way, it was possible for the group to interact and to collect quantitative data as well.

The interview was conducted and moderated by the first author and co-conducted by the second author, while the third author took notes. The focus group was designed as a semi-structured interview. It aimed to investigate participants′ beliefs about the necessity of animal protein in nutrition, particularly among individuals who engage in physical activity. We used the Mentimeter interface to create interactive word clouds that captured the participants′ opinions on the positive and negative aspects of adopting a plant-based diet. Additionally, the discussion explored attitudes and stereotypes surrounding plant-based protein foods and individuals who adhere to a strictly plant-based diet. A series of adjectives was presented via the Mentimeter interface, enabling participants to indicate their levels of agreement or disagreement. The complete trace and procedural details of the focus group and examples of arguments from participants are available in the Supplementary Materials.

### 2.3. Data Analysis

The information obtained from the four focus groups was analyzed using thematic analysis, following the procedures outlined by Braun and Clarke [44]. The audio recording was transcribed in full, ensuring anonymity. Thematic analysis was conducted separately and independently by the first three authors by following a 6-step procedure: 1. Familiarization with the data; 2. Generation of initial codes; 3. Searching for themes; 4. Review of themes; 5. Definition and naming of themes; 6. Report writing. Then, the researchers compared and integrated their respective outcomes, ensuring a collaborative reflection on the themes to enrich data interpretation. An integration of the deductive and inductive approaches was applied; themes were identified in relation to the research questions, and both the manifest and latent contents that emerged in the discussion were taken into consideration. Anonymized quotations were selected to illustrate the results of the thematic analysis and to support the discussion.

The items of the quantitative sections were created using a semantic differential, in other words, expressing two opposite polarities. Responses ranged from 1 (e.g., “bland”) to 10 (e.g., “tasty”); see the Supplementary Materials for a complete description of the items. Responses were analyzed using a series of robust two-factorial ANOVAs [45] as implemented in Jamovi (Version 2.3.28, The Jamovi project, 2022, retrieved from https://www.jamovi.org). The attitudes were the dependent variables, while dietary regimen (two levels: veg*an vs. omnivore) and physical activity levels (sedentary vs. active) were the independent variables. All statistical tests were two-tailed, and a *p* ≤ 0.05 was considered statistically significant.

## 3. Results and Discussion

The first research question—–Does physical activity represent a barrier to plant-based choices?”—was addressed through a deductive framework. Drawing on the existing literature, we anticipated the presence of attitudinal biases and resistance, primarily linked to physical activity and secondarily to dietary style. To investigate this, targeted questions were formulated to guide the focus group discussions (see Section 2.2, Part II). The resulting themes for Research Question I are presented in Section 3.1.

The second research question examined additional factors that may act as either barriers or facilitators of plant-based dietary choices and how these factors vary across different lifestyle groups. Given the exploratory nature of this aim, an inductive approach was adopted. Themes were derived from open-ended and interactive segments of the group discussions. The findings related to this question are outlined in Section 3.2 (barriers) and Section 3.3 (facilitators), with results disaggregated by dietary pattern and level of physical activity when applicable.

Finally, in addition to qualitative thematic coding, participants′ responses to the interactive survey were analyzed. These results are reported in Section 3.4 and provide additional context for interpreting group-level attitudes.

### 3.1. Research Question I: Does Physical Activity Represent a Barrier to Plant-Based Choice?

#### 3.1.1. Theme 1: Comparing the Effectiveness of Animal and Plant-Based Proteins for Performance

The groups of omnivores were inclined to frequently frame meat consumption as essential for athletic performance (Table 2). The sedentary group expressed some doubts and placed greater emphasis on the importance of professional guidance during dietary transitions.

Veg*ans strongly advocated for the nutritional viability of plant-based proteins in sustaining athletic performance (Table 2). Moreover, plant proteins provided the perception of greater strength and energy. The active group, in particular, reported on the theme.

Thus, physical activity plays a crucial role in influencing attitudes towards diet, particularly regarding the source of protein. In the case of omnivores, this finding aligns with previous literature associating meat with strength and performance [33]. In the case of veg*ans, the increased sense of energy may be interpreted through the lens of the mastery experience construct [46]. Thus, the perceived ability to “make it” appears to be externally attributed—linked to the power of meat—among omnivores. In contrast, for veg*ans, it is internally attributed, grounded in their own abilities. This theme is further elaborated in Section 3.2.4, which discusses inner strength.

#### 3.1.2. Theme 2: Sources of Knowledge

Among active omnivores, the influence of professional figures—such as physicians, coaches, and athletic trainers—emerged as a significant factor in shaping nutritional beliefs (Table 2), and in conveying the message that plant-based diets are nutritionally inadequate for physically active individuals. In contrast, no explicit reference to professional figures emerged in the case of sedentary omnivores, who justified their habits by referencing the Mediterranean diet as a general framework for balanced eating. They emphasized the importance of “eating a bit of everything,” often highlighting their habit of consuming only small amounts of meat. These internalized guidelines, deeply rooted in the Italian sociocultural matrix, suggest that although Mediterranean cuisine inherently offers many plant-based options, a stronger drive to adhere to culinary traditions remains, which may act as an additional barrier to dietary change. This finding aligns with previous literature, which identifies a strong sense of belonging to a culinary background as a key factor limiting openness to novelty and dietary innovation [47,48].

The role of professionals was also present in the veg*an groups, but there was no mention of coaches, only of physicians and nutritionists (Table 2). Similarly to the active omnivore group, the theme of the role of professionals was related to planning a balanced diet, with the proper protein intake. However, there was no bias against a plant-based diet in this case, probably because participants contacted vegan or open-to-vegan nutritionists. In some cases, different sources of information, such as the internet, TV movies/documentaries, or social media, were reported, even if they were more prevalent in the sedentary group.

This pattern may be explained by the differential role of perceived authoritative sources in shaping and maintaining dietary attitudes. Among omnivores, trust tends to be placed in traditional figures (e.g., physicians and coaches), to whom nutritional expertise is delegated. In contrast, veg*ans appear to rely on sources perceived as more up-to-date, open-minded, and aligned with their personal values. Previous literature has shown that veg*ans′ experiences with healthcare professionals are not always positive, as they often perceive stigma or a lack of understanding regarding their specific needs and requirements [49]. Consequently, information is acquired independently, often online and through specialized publications [49]. Consistent with this pattern, previous studies have reported that flexitarians and veg*ans score higher on measures of general nutrition knowledge and diet quality [50].

### 3.2. Research Question II: What Are Other Barriers to Plant-Based Choice? How Do They Vary by Lifestyle?

#### 3.2.1. Theme III: Social Stigma

All groups acknowledged that adopting a plant-based diet may present social challenges, particularly in the Italian culture, where food is closely tied to conviviality and shared identity.

In the omnivore groups, sedentary participants dwelled on the lack of support. Active omnivores, on the other hand, believed that a vegan diet is more exclusive and, consequently, can lead to social isolation (Table 3). Both groups only mentioned this aspect, which was not explored in depth.

In contrast, veg*an participants shared personal and emotionally salient accounts of social stigma. Both active and sedentary groups referred to conflict—particularly within family contexts—and feelings of being misunderstood or judged (Table 3). These findings align with those of Markowski and Roxburgh [51], who found that non-vegans anticipated stigma and social rejection as barriers to adopting a plant-based lifestyle, leading to subsequent social and behavioral distancing.

This theme clearly illustrates how dietary choices intersect with identity issues that distinguish ingroup and outgroup members. Adherence to a vegan or vegetarian diet can function as a social identity marker, which, however, may generate dissonance and intergroup bias when it clashes with dominant cultural norms, such as those related to conviviality and the Mediterranean dietary tradition. Omnivores often describe veganism as “exclusive,” whereas veg*ns report experiences of exclusion and misunderstanding (e.g., jokes at the table, family conflicts). In summary, eating practices can become symbolic boundaries between groups, carrying implications of belonging, differentiation, and social tension [15].

#### 3.2.2. Theme IV: Renunciation

The theme of renunciation—the perceived loss of taste, satisfaction, or familiarity—was identified across all four groups, regardless of dietary pattern or activity level, but more prominently in the omnivore active group. Omnivores focused mainly on the difficulty of giving up meat. Veg*ans, instead, reported their difficulties in giving up cheese. However, they also extend the theme beyond taste to social life, as already mentioned in the previous paragraph (Table 3).

This theme is closely intertwined with personal values, particularly that of hedonism. Among omnivores, giving up the taste of meat or certain flavors is perceived as the loss of a legitimate source of pleasure. Previous research has conceptualized this resistance through the lens of cognitive and sociocultural biases that position meat consumption as the default or normative choice [6,15,52,53]. In the present study, however, the sense of loss was not primarily related to tradition or maintaining the status quo, but rather to the sensory gratification associated with meat. This finding is consistent with prior research, which has shown that, beyond the reluctance to change eating habits, the enjoyment of eating meat is one of the main perceived barriers to adopting a vegetarian diet [52]. This distinction is valuable for future research aiming to disentangle the motivational factors underlying dietary preferences. Among veg*an participants, the theme of pleasure was acknowledged but subordinated to other guiding values, such as health, animal compassion, and sustainability, which are the most frequent promoters of plant-based choices [47] (see also Section 3.3)

#### 3.2.3. Theme V: Logistics and Organization

Logistical and practical barriers emerged as relevant across all groups. Participants identified cooking skills, time management, and food accessibility as central challenges in maintaining a plant-based diet (Table 3). Omnivores highlighted organizational difficulties, particularly in households with mixed diets or in work environments where vegan options are limited. A recurring concern was the belief that plant-based diets require advanced cooking skills to ensure flavor and nutritional adequacy. This theme is intertwined with that of renunciation. Because the plant-based diet contains fewer ingredients, it requires cooking skills to be tasty.

Among veg*ans, logistical barriers were mainly linked to food availability, especially when eating outside the home or living in smaller towns.

The emergence of this theme aligns with findings from a recent review, which described veg*an diets as often perceived as inconvenient and effortful [54]. Additionally, it emphasizes the importance of perceptions of self-efficacy and organizational skills in adopting a plant-based diet. These abilities are influenced by both the value of autonomy (self-direction), reflected in the capacity to plan meals and select appropriate recipes, and the pursuit of a personal routine that satisfies the need for stability and control (security). Self-direction and security have also been identified in previous research as important motivational values for adopting and maintaining a vegan diet [55].

#### 3.2.4. Theme VI: Inner Strength

Closely related to the theme of renunciation, a final theme that emerged across most groups was the perception that inner strength or psychological resilience is required to maintain a plant-based diet, particularly in the face of social and cultural pressures. Regarding omnivores, this theme was expressed by acknowledging that adopting and maintaining a vegan diet requires self-discipline, consistency, and mental effort (Table 4). This notion was further emphasized by the veg*an groups, who referred to themes of consciousness and courage. Thus, the inner strength required to adopt and maintain a plant-based diet appears to stem not merely from self-discipline or willpower, but from a form of moral resilience grounded in acting consistently with one’s moral beliefs, in line with the theory of moral conviction [56].

**Figure 1:**
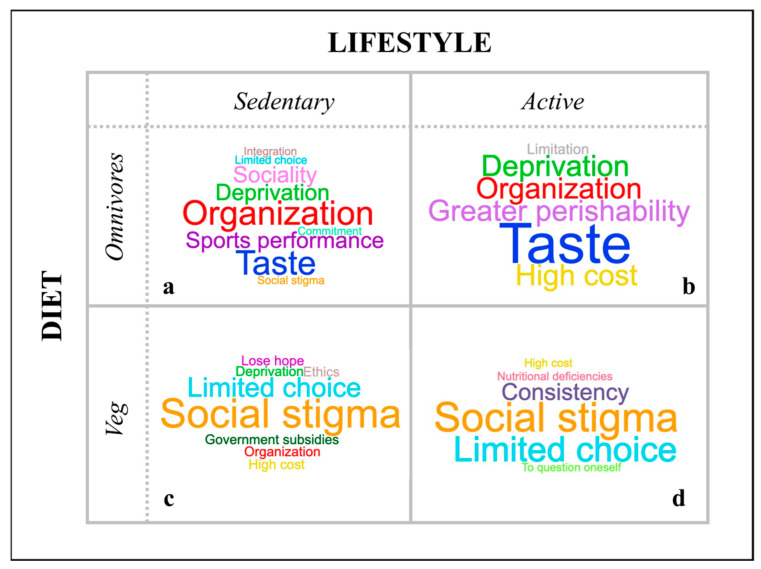
Word clouds summarizing participants’ responses to the question: “Imagine starting a totally plant-based diet. What would be the negative aspects? Write down as many words as you can think of.” Above are illustrated the word clouds generated by the sedentary omnivorous participants (section (**a**)) and the active omnivorous participants (section (**b**)). Below are illustrated the word clouds generated by the sedentary veg*an participants (section (**c**)) and the active omnivorous participants (section (**d**)).

Figure 1 illustrates the responses of the four groups regarding the negative aspects of plant-based choices.

### 3.3. Research Question II: What Are the Promoters of the Plant-Based Choice? How Do They Vary by Lifestyle?

#### 3.3.1. Theme VII: Ethics

Ethical considerations were mentioned only marginally by omnivore groups, which acknowledged the potential benefits of plant-based diets for animal welfare but did not elaborate or engage in in-depth discussion.

In contrast, veg*an participants consistently identified ethical concerns as a central reason for adopting a plant-based diet. Ethical motivations were often linked to powerful emotional experiences, primarily upon exposure to media content depicting animal suffering in industrial farming settings.

Whereas in the narrative of omnivores this issue was approached in a synthetic/detached way, in the case of veg*ans, there was a strong emotional involvement. The ethical discourse extended beyond opposition to animal cruelty, encompassing broader principles of respect for life and a desire to avoid complicity in systemic exploitation. Participants described a sense of inner peace resulting from the alignment between their values and dietary choices (Table 3).

This theme further underscores the central role of values in guiding the adoption of a plant-based diet—particularly: respect for life, compassion toward animals, and the desire to reduce the suffering of others. Such choices are therefore primarily driven by ethical motivations, accompanied by strong emotional engagement that strengthens the alignment between individual behavior and the value of universalism [47].

#### 3.3.2. Theme VIII: Health

Health was mentioned as a potential benefit of plant-based diets by both omnivore subgroups. Sedentary omnivores focused on digestive health, describing plant-based foods as lighter and more beneficial for gut flora, which they associated with general well-being and immune function. Besides acknowledging some health benefits, active omnivores tended to emphasize the lower fat content and lighter preparation methods of plant-based dishes.

On the other hand, veg*an participants described plant-based diets as the healthiest available option, often referring to scientific evidence or public discourse linking meat consumption to major diseases. Some participants—particularly in the sedentary group—also connected the theme of health with ethical concerns. They argued that food derived from suffering animals cannot be healthy, reflecting a holistic view that blends physical, emotional, and ethical dimensions of health (Table 4).

Similarly to the theme of “sources of knowledge”, the omnivore groups once again showed a tendency to rely on shared cultural beliefs—particularly the recommendations of the Mediterranean diet. In contrast, the veg*an groups displayed a more substantial interest in continuous learning and up-to-date information. The latter also appeared more aware of the health risks associated with meat and animal product consumption, such as colorectal cancer and cardiovascular diseases. Recent studies have indeed demonstrated higher levels of general nutrition knowledge among these groups, which can support this finding [50].

#### 3.3.3. Theme IX: Sustainability

Omnivores were not very vocal about the issue related to environmental sustainability, and they only mentioned it with no further elaboration. Among veg*an participants, sustainability emerged as a well-developed and frequently cited motivator. Participants demonstrated extensive knowledge about this issue (Table 4).

This theme, along with the previous two, has been identified in the literature as one of the primary motivators underlying the adoption of a plant-based diet [21,57]. Veg*an participants, in particular, appeared to perceive their personal role in contributing to collective change—as previously noted [56], for instance, by reducing emissions or mentioning the potential to address global hunger. They demonstrated awareness of the connection between individual choices and global impact, which serves as a strong driver of sustainable behaviors. These findings once again emphasize the central role of personal values, particularly the dimension of universalism, which has been consistently linked to plant-based dietary choices [47].

**Figure 2:**
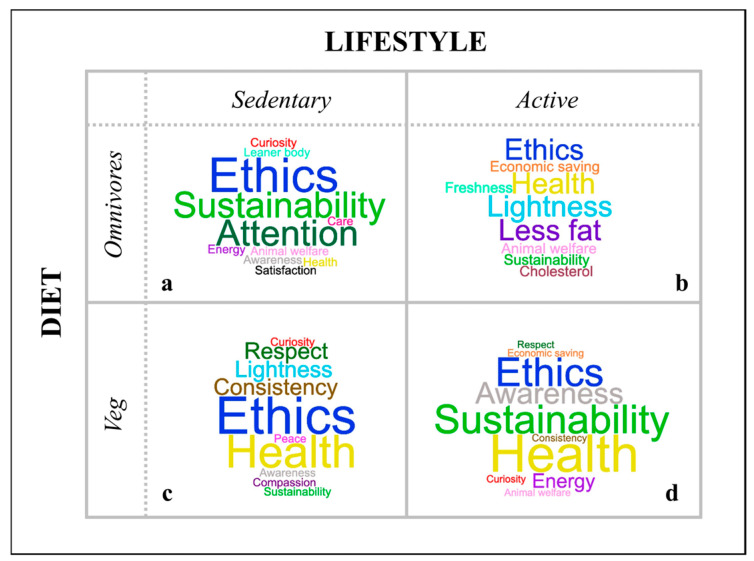
Word clouds summarizing participants’ opinions in response to the question: "Imagine starting a totally plant-based diet. What would be the positive aspects? Write down as many words as you can think of." The word clouds generated by the sedentary omnivorous participants are illustrated above (section (**a**)), alongside those from the active omnivorous participants (section (**b**)). Below are the word clouds created by the sedentary vegan participants (section (**c**)) and the active vegan participants (section (**d**)).

Figure 2 illustrates the responses of the four groups regarding the positive aspects of plant-based choices.

### 3.4. Interactive Survey: Attitude Towards Plant-Based Protein Dishes and People Following a Fully Plant-Based Diet

To complement the qualitative data, participants also completed a brief interactive survey via Mentimeter during the focus group sessions. The survey aimed to capture attitudes toward both plant-based protein dishes and individuals who adhere to an entirely plant-based diet. While the primary purpose of these items was to stimulate discussion, the resulting responses offered additional insight into group-level perceptions.

The results suggested that when asked about their thoughts on plant-based protein dishes, the omnivore groups rated plant-based protein food more negatively than the veg*an groups in all aspects except healthiness (Table 5). The dimensions on which the two groups differed the most were difficult/easy, inferior/superior, and exclusive/inclusive, in which the omnivore groups expressed more negative attitudes than the veg*an ones.

In response to the question, “Thinking about people who follow a fully plant-based diet, how would you rate them?”, omnivore groups exhibited a higher level of negative bias, assigning the lowest scores across the dimensions weak/strong and pale/rose (Table 6). Across all groups, a tendency to associate veganism with femininity was observed. However, a significant interaction emerged between dietary habits and sports participation. Active omnivores referred to vegans as “female” more frequently than sedentary omnivores. The reverse occurred among veg*an groups: sedentary veg*ans referred to vegans as “female” more frequently than active veg*ans. This group associated plant-based diets with femininity more often in the anonymous survey. This finding, extensively discussed in the literature in relation to sport and anticipated in our hypotheses, suggests a potential stigma that fits within the theoretical framework of hegemonic masculinity and may extend to attitudes toward plant-based choices [33].

## 4. Conclusions

The present study was guided by two main research questions: I) whether and how physical activity may act as a barrier to adopting a plant-based diet, and II) the presence of additional facilitating or impeding factors, with attention to differences across lifestyle groups.

In relation to the first research question, and consistent with our hypotheses, the findings suggest that physical activity may amplify the existing stigma associated with plant-based diets. The physically active omnivore group, in particular, emphasized the centrality of meat in sustaining athletic performance, reinforced by the beliefs of professional figures such as coaches and trainers. These convictions—already reported in previous studies [33]—were primarily justified by nutritional concerns. This point is particularly relevant, as omnivore participants reported relying on traditional authorities (e.g., physicians, trainers) or internalized norms related to the Mediterranean diet as their primary sources of nutritional guidance. Consequently, they appeared less proactive in seeking nutritional information compared to veg*an participants, who demonstrated higher levels of knowledge on these topics. In this context, the Mediterranean diet may represent a deeply rooted traditional model that, despite its compatibility with plant-based choices, acts as a cultural barrier to dietary change. This finding aligns with previous research, which identifies a strong sense of attachment to culinary heritage as a factor limiting openness to novelty and dietary innovation [47,48].

Another result consistent with our hypotheses concerns the theme of masculinity, which this group more prominently mentioned in the anonymous survey, where plant-based diets were more frequently associated with femininity. This finding, widely discussed in the literature on sport, suggests a potential stigma fitting within the theoretical framework of hegemonic masculinity that may extend to attitudes toward plant-based choices [33].

Moreover, active omnivores referenced the pleasure of eating meat more frequently, suggesting the role of hedonistic values. In line with this interpretation, they acknowledged the health-related benefits of plant-based diets but primarily framed them in terms of lightness and low-fat content—associations more aligned with body image and fitness ideals (and gendered expectations) than with actual nutritional adequacy. Hence, physically active individuals may perceive greater losses when giving up meat, both in terms of nutrition and symbolism. This evidence points to a potential interaction among physical activity, aesthetic ideals, gender norms, and dietary restraint—an avenue for further exploration in future work.

Regarding the second research question, three key motivational drivers for adopting plant-based diets emerged: ethics, health, and sustainability, consistent with previous literature that describes them as a recurring triad [21,57]. However, while omnivores briefly mentioned these aspects, veg*an participants expressed them with greater emotional intensity. The only notable distinction between active and sedentary omnivores concerned the theme of health: for sedentary participants, plant-based diets were described as digestible, whereas active participants viewed them as a dietetic alternative with low fat content. This evidence reflects both the perception of plant proteins as nutritionally insufficient for performance and, more subtly, the gendered association between dietary restraint and masculinity.

Regarding perceived barriers to a plant-based transition, participants mentioned the renunciation of taste and tradition, organizational difficulties, fear of social stigma, and the need for inner strength. These themes were discussed most passionately by veg*an groups, who also reported personal experiences of exclusion and misunderstanding. No substantial differences emerged in relation to physical activity across dietary groups, except for the theme of renunciation, which was more prevalent among active omnivores—again tied to hedonistic considerations.

It is important to acknowledge that certain methodological limitations may restrict the generalizability of these findings. The sample size was relatively limited compared to most previous studies. The number of focus groups—constrained by the difficulty of recruiting veg*an participants, particularly men—was pre-determined, and data robustness was evaluated a posteriori. The sample size is aligned with the information power framework [58] and comparable to some previous qualitative studies within this field [51,59]. Also, the quality of our data was further strengthened by triple independent coding and mixed-methods analyses. However, this choice represents a potential limitation and should be addressed in future investigations.

Also, the veg*an sample included a higher proportion of vegans compared to vegetarians. Although the analyses focused on the broader contrast between omnivores and veg*ans, aligning comparable themes across sub-groups (e.g., meat vs. cheese renunciation), this imbalance may hold particular relevance when interpreting the results within the theoretical framework of hegemonic masculinity, which is mainly associated with meat consumption rather than other animal-derived products. For these reasons, future research may benefit from distinguishing more clearly between vegan and vegetarian participants.

Moreover, the influence of other socio-demographic variables, such as education level and socioeconomic status, should also be addressed to broaden the discussion in relation to specific themes such as sources of knowledge, renunciation, and organization. The same applies to age. The sample included only individuals aged 18–45, in line with prior research suggesting that younger adults are both more likely to consume meat and more open to behavior change [20,60]. This population was chosen as a target group for potential educational interventions. However, given the central role of values in the present findings, future research could benefit from comparing older and younger adults to generate more specific insights.

Finally, the role of sports warrants further investigation, particularly through comparisons between amateur and professional athletes, given the influence of coaches and institutional norms on dietary choices.

Despite these limitations, the study provides valuable insights into the connections between representations of physical activity and attitudes toward plant-based diets. The focus on the Italian context and its specific dietary habits offers a culturally grounded perspective that may inform future educational interventions aimed at promoting a positive view of plant-based eating—one that emphasizes enrichment and adherence to traditional ingredients [42], rather than deprivation. In particular, within sports and physical activity contexts, these findings underscore the need to cultivate a new narrative that challenges existing biases about dietetic alternatives and reframes plant-based diets as compatible with strength, vitality, and overall well-being. This new framework could promote greater acceptance of plant-based diets among diverse populations, particularly those more closely tied to athletic identity and performance.

## Figures and Tables

**Table 1:** Characteristics of the participants (*n* = 26).

Diet	Omnivores		Veg*ans	
Physical Activity	Sedentary	Active	Sedentary	Active
Sample size	*n* = 7	*n* = 7	vegetarian: *n* = 1 vegans: *n* = 5	vegetarians: *n* = 2 vegans: *n* = 4
Gender	♂ = 3; ♀ = 3; NB = 1	♂ = 3; ♀ = 4	♂ = 2; ♀ = 4	♂ = 2; ♀ = 4
Age range	25–33	26–35	19–44	27–35
Mean age and SD	M = 28.14; SD = 3.07	M = 29.85; SD = 3.13	M = 30.33; SD = 10.05	M = 30.66; SD = 3.26
Sports	None or occasional	Soccer: *n* = 1 Athletics: *n* = 2 Rock climbing: *n* = 2 Kickboxing: *n* = 1 Tchoukball: *n* = 1	None or occasional	Running: *n* = 2 Calisthenics: *n* = 1 Trail: *n* = 1 Shaolin: *n* = 1 Workout: *n* = 1 Trekking: *n* = 2 Rock climbing: *n* = 1

Note. ♂ = men; ♀ = women; NB = non binary

**Table 2:** Themes related to the role of physical activity in modulating attitudes towards plant-based sources (Research Question I).

RQ I	The Role of Physical Activity in Modulating Attitudes Towards Plant-Based Sources			
**Theme I**	**Comparing the effectiveness of animal and plant proteins for performance.**			
**Group**	**Omn Sed**	**Omn Act**	Veg*an Sed	Veg*an Act
	Doubts, importance of being advised by a professional	Animal protein is essential for sports	Importance of being advised by a professional	Animal protein not essential; plant-based protein more energetic
**Theme II**	**Sources of knowledge**			
**Group**	**Omn Sed**	**Omn Act**	Veg*an Sed	Veg*an Act
	Tradition	Physicians; coaches	Internet; documentaries; social media	Physicians; nutritionists; videos

**Table 3:** Themes related to the cons of plant-based choice (Research Question II).

RQ II	The Cons of Plant-Based Choice			
**Theme III**	**Social stigma**			
**Group**	**Omn Sed**	**Omn Act**	Veg*an Sed	Veg*an Act
	Lack of support/services	Exclusivity/social isolation	Feeling of being misunderstood	Fear of becoming a burden
**Theme IV**	**Renunciation**			
**Group**	**Omn Sed**	**Omn Act**	Veg*an Sed	Veg*an Act
	Attachment to meat flavor		Nostalgia for cheese taste	
**Theme V**	**Logistics and organization**			
**Group**	**Omn Sed**	**Omn Act**	Veg*an Sed	Veg*an Act
	High culinary skills to match meat flavors		Difficulties in finding vegan options at restaurants	
**Theme VI**	**Inner strength**			
**Group**	**Omn Sed**	**Omn Act**	Veg*an Sed	Veg*an Act
	Difficulty in resisting temptations	-	Tenacity in being an outsider	Consciousness and courage

**Table 4:** Themes related to the pros of plant-based choice.

RQ II	The Pros of Plant-Based Choice			
**Theme VII**	**Ethics**			
**Group**	**Omn Sed**	**Omn Act**	Veg*an Sed	Veg*an Act
	Just mentioned		Strong emotional drive	
**Theme VII**	**Health**			
**Group**	**Omn Sed**	**Omn Act**	Veg*an Sed	Veg*an Act
	Healthier, better digestion, gut health	Lighter, low-fat	The way animals are treated makes meat unhealthy	Prevention of cancer, cardiovascular diseases
**Theme IX**	**Sustainability**			
**Group**	**Omn Sed**	**Omn Act**	Veg*an Sed	Veg*an Act
	Just mentioned		Very knowledgeable	

**Table 5:** Results of the quantitative data. Participants answer the following question: “Thinking about plant-based protein dishes, such as the ones we just discussed, how would you rate them?” The response scale ranged from 1 (e.g., Bland) to 10 (e.g., Tasty).

	Median					
	Omn Sed	Omn Act	Veg*an Sed	Veg*an Act	Significant Effect	*p* Value
(1) Bland … (10) Tasty	7.0	6.0	8.0	9.0	Diet	0.025
(1) Poor… (10) Nutritious	8.0	8.0	10.0	9.5	Diet	0.015
(1) Unhealthy … (10) Healthy	9.0	8.0	10.0	9.0	/	
(1) Inferior … (10) Superior	5.5	5.0	10.0	9.5	Diet	<0.001
(1) Unsatisfying … (10) Fulfilling	6.5	4.0	7.0	9.5	Diet	0.005
(1) Difficult … (10) Easy	3.5	4.0	9.0	9.0	Diet	<0.001
(1) Boring … (10) Various	6.5	6.0	8.0	10.0	Diet	0.019
(1) Exclusive … (10) Inclusive	3.0	5.0	9.0	7.5	Diet	0.002

Note. The significant effect of Diet suggests, for example, that omnivore groups rated plant-based dishes as blander than veg*an groups.

**Table 6:** Results of the quantitative data. Participants answer the following question: “Thinking about people who follow a fully plant-based diet, how would you rate them?” The response scale ranged from 1 (e.g., Weak) to 10 (e.g., Strong).

	Median					
	Omn Sed	Omn Act	Veg*an Sed	Veg*an Act	Significant Effect	*p* Value
(1) Sad … (10) Happy	5.5	8.0	8.0	9.0	/	
(1) Weak … (10) Strong	5.0	8.0	10.0	10.0	Diet	<0.001
(1) Male … (10) Female	5.0	8.0	8.0	5.0	Diet × Sport	<0.001
(1) Pale … (10) Rosy	5.0	5.0	7.0	8.0	Diet	0.006
(1) Flabby … (10) Muscular	5.0	6.0	6.0	6.0	/	

Note. The significant effect of Diet suggests, for example, that omnivore groups refer to vegans as “weak” more frequently than veg*an groups.

## Data Availability

The original contributions presented in the study are included in the article/Supplementary Materials, further inquiries can be directed to the corresponding author.

## Supplementary Materials

The following supporting information can be downloaded at: https://www.mdpi.com/article/10.3390/nu17213362/s1, File S1: Focus group outline; Table S1: Main themes emerging from the focus groups and examples of arguments produced by the participants.

## Abbreviations

The following abbreviations are used in this manuscript:

Omn	Omnivore participants
Veg*an	Vegetarian and vegan participants

## References

[1] Foley J.A., Ramankutty N., Brauman K.A., Cassidy E.S., Gerber J.S., Johnston M., Mueller N.D., O’Connell C., Ray D.K., West P.C. (2011). Solutions for a Cultivated Planet. Nature.

[2] Ganivet E. (2020). Growth in Human Population and Consumption Both Need to Be Addressed to Reach an Ecologically Sustainable Future. Environ. Dev. Sustain..

[3] Food and Agriculture Drganization of the United Nations (2022). FAO Greenhouse Gas Emissions from Agrifood Systems.

[4] Xu X., Sharma P., Shu S., Lin T.-S., Ciais P., Tubiello F.N., Smith P., Campbell N., Jain A.K. (2021). Global Greenhouse Gas Emissions from Animal-Based Foods Are Twice Those of Plant-Based Foods. Nat. Food.

[5] Herrero M., Wirsenius S., Henderson B., Rigolot C., Thornton P., Havlík P., De Boer I., Gerber P. (2015). Livestock and the Environment: What Have We Learned in the Past Decade?. Annu. Rev. Environ. Resour..

[6] Donato C., Monsurrò L., Di Cioccio M. (2024). A Matter of Identity: Promoting Plant-Based Food among Meat-Eaters through a Common Identity Priming. J. Retail. Consum. Serv..

[7] Mouat M.J., Prince R., Roche M.M. (2019). Making Value Out of Ethics: The Emerging Economic Geography of Lab-Grown Meat and Other Animal-Free Food Products. Econ. Geogr..

[8] Boy E., Brouwer I., Foley J., Palacios N., Scott S., Taleon V. (2024). Global Food Policy Report 2024: Leveraging Plant-Source Foods for Sustainable Healthy Diets.

[9] Fehér A., Gazdecki M., Véha M., Szakály M., Szakály Z. (2020). A Comprehensive Review of the Benefits of and the Barriers to the Switch to a Plant-Based Diet. Sustainability.

[10] Pollakova D., Andreadi A., Pacifici F., Della-Morte D., Lauro D., Tubili C. (2021). The Impact of Vegan Diet in the Prevention and Treatment of Type 2 Diabetes: A Systematic Review. Nutrients.

[11] Tantamango-Bartley Y., Jaceldo-Siegl K., Fan J., Fraser G. (2013). Vegetarian Diets and the Incidence of Cancer in a Low-Risk Population. Cancer Epidemiol. Biomark. Prev..

[12] Barnard N.D., Alwarith J., Rembert E., Brandon L., Nguyen M., Goergen A., Horne T., do Nascimento G.F., Lakkadi K., Tura A. (2022). A Mediterranean Diet and Low-Fat Vegan Diet to Improve Body Weight and Cardiometabolic Risk Factors: A Randomized, Cross-over Trial. J. Am. Nutr. Assoc..

[13] Williams E., Vardavoulia A., Lally P., Gardner B. (2023). Experiences of Initiating and Maintaining a Vegan Diet among Young Adults: A Qualitative Study. Appetite.

[14] Meier T., Christen O. (2013). Environmental Impacts of Dietary Recommendations and Dietary Styles: Germany As an Example. Environ. Sci. Technol..

[15] Rosenfeld D.L., Rothgerber H., Tomiyama A.J. (2024). When Meat-Eaters Expect Vegan Food to Taste Bad: Veganism as a Symbolic Threat. Group Process. Intergroup Relat..

[16] Eurispes 36mo Rapporto Italia—Percorsi Di Ricerca Nella Società Italiana [36th Italian Report—Research Paths in Italian Society]; 2024. https://eurispes.eu/wp-content/uploads/2024/05/2024_rapporto_italia_sintesi.pdf.

[17] MacInnis C.C., Hodson G. (2017). It Ain’t Easy Eating Greens: Evidence of Bias toward Vegetarians and Vegans from Both Source and Target. Group Process. Intergroup Relat..

[18] Adams C.J. (2015). The Sexual Politics of Meat.

[19] Bagci S.C., Rosenfeld D.L., Uslu D. (2021). Intergroup Attitudes between Meat-Eaters and Meat-Avoiders: The Role of Dietary Ingroup Identification. Group Process. Intergroup Relat..

[20] Monteiro B.M.A., Pfeiler T.M., Patterson M.D., Milburn M.A. (2017). The Carnism Inventory: Measuring the Ideology of Eating Animals. Appetite.

[21] Ruby M.B. (2012). Vegetarianism. A Blossoming Field of Study. Appetite.

[22] Rosenfeld D.L. (2018). The Psychology of Vegetarianism: Recent Advances and Future Directions. Appetite.

[23] English C. (2017). Toward Sport Reform: Hegemonic Masculinity and Reconceptualizing Competition. J. Philos. Sport.

[24] Mertens A., Oberhoff L. (2023). Meat-Eating Justification When Gender Identity Is Threatened—The Association between Meat and Male Masculinity. Food Qual. Prefer..

[25] Ramaeker J., Petrie T.A. (2019). “Man up!”: Exploring Intersections of Sport Participation, Masculinity, Psychological Distress, and Help-Seeking Attitudes and Intentions. Psychol. Men Masculinities.

[26] Vartanian L.R. (2015). Impression Management and Food Intake. Current Directions in Research. Appetite.

[27] De Groeve B., Rosenfeld D.L. (2022). Morally Admirable or Moralistically Deplorable? A Theoretical Framework for Understanding Character Judgments of Vegan Advocates. Appetite.

[28] Graça J., Calheiros M.M., Oliveira A., Milfont T.L. (2018). Why Are Women Less Likely to Support Animal Exploitation than Men? The Mediating Roles of Social Dominance Orientation and Empathy. Personal. Individ. Differ..

[29] Judge M., Wilson M.S. (2019). A Dual–process Motivational Model of Attitudes towards Vegetarians and Vegans. Eur. J. Soc. Psychol..

[30] Kårlund A., Gómez-Gallego C., Turpeinen A.M., Palo-oja O.-M., El-Nezami H., Kolehmainen M. (2019). Protein Supplements and Their Relation with Nutrition, Microbiota Composition and Health: Is More Protein Always Better for Sportspeople?. Nutrients.

[31] Antonio J., Peacock C.A., Ellerbroek A., Fromhoff B., Silver T. (2014). The Effects of Consuming a High Protein Diet (4.4 g/Kg/d) on Body Composition in Resistance-Trained Individuals. J. Int. Soc. Sports Nutr..

[32] Clark A., Mach N. (2016). Exercise-Induced Stress Behavior, Gut-Microbiota-Brain Axis and Diet: A Systematic Review for Athletes. J. Int. Soc. Sports Nutr..

[33] van der Horst H., Sällylä A., Michielsen Y. (2023). Game Changers for Meat and Masculinity? Male Athletes’ Perspectives on Mixed and Plant-Based Diets. Appetite.

[34] Di Corcia M., Tartaglia N., Polito R., Ambrosi A., Messina G., Francavilla V.C., Cincione R.I., Malva A.d., Ciliberti M.G., Sevi A. (2022). Functional Properties of Meat in Athletes’ Performance and Recovery. Int. J. Environ. Res. Public Health.

[35] Close G.L., Hamilton D.L., Philp A., Burke L.M., Morton J.P. (2016). New Strategies in Sport Nutrition to Increase Exercise Performance. Free Radic. Biol. Med..

[36] Devries M.C., Phillips S.M. (2015). Supplemental Protein in Support of Muscle Mass and Health: Advantage Whey. J. Food Sci..

[37] Craddock J.C., Probst Y.C., Peoples G.E. (2016). Vegetarian and Omnivorous Nutrition—Comparing Physical Performance. Int. J. Sport Nutr. Exerc. Metab..

[38] Lynch H., Johnston C., Wharton C. (2018). Plant-Based Diets: Considerations for Environmental Impact, Protein Quality, and Exercise Performance. Nutrients.

[39] Roberts A.K., Busque V., Robinson J.L., Landry M.J., Gardner C.D. (2022). SWAP-MEAT Athlete (Study with Appetizing Plant-Food, Meat Eating Alternatives Trial)—Investigating the Impact of Three Different Diets on Recreational Athletic Performance: A Randomized Crossover Trial. Nutr. J..

[40] Colombo M. (1997). Il Gruppo Come Strumento Di Ricerca Sociale: Dalla Comunità Al Focus Group. Studi Sociol..

[41] Chironi S., Bacarella S., Altamore L., Columba P., Ingrassia M. (2021). Consumption of Spices and Ethnic Contamination in the Daily Diet of Italians—Consumers’ Preferences and Modification of Eating Habits. J. Ethn. Foods.

[42] Adorni R., Vanutelli M.E., Mammano A., Cambieri V.C., D’Addario M., Steca P. (2026). Not Only Veggies: Promoting Healthy and Familiar Plant-Based Alternatives to Traditional Meat Dishes with the AttiFood Tool. Food Qual. Prefer..

[43] Barrena R., Sánchez M. (2009). Consumption Frequency and Degree of Abstraction: A Study Using the Laddering Technique on Beef Consumers. Food Qual. Prefer..

[44] Braun V., Clarke V. (2006). Using Thematic Analysis in Psychology. Qual. Res. Psychol..

[45] Wilcox R.R. (2011). Introduction to Robust Estimation and Hypothesis Testing.

[46] Felttz D.L., Short S.E., Sullivan P.J. (2008). Self-Efficacy in Sport.

[47] Lehto E., Korhonen K., Muilu T., Konttinen H. (2023). How Do Values Relate to the Consumption of Meat and Dairy Products and Their Plant-Based Alternatives?. Food Qual. Prefer..

[48] Vanutelli M.E., Adorni R., Leone P.A., Luperini A., D’Addario M., Steca P. (2025). Never Too Late to Try Something New. Attitudes and Intention to Taste Foods from Alternative Protein Sources in a Sample of Italian Older Adults. Front. Sustain. Food Syst..

[49] Jirovsky-Platter E., Wakolbinger M., Kühn T., Hoffmann K., Rieder A., Haider S. (2024). Experiences of Vegans with General Practitioners in the Austrian Health Care System: A Qualitative Study. Nutrients.

[50] Groufh-Jacobsen S., Larsson C., Van Daele W., Margerison C., Mulkerrins I., Aasland L.M., Medin A.C. (2023). Food Literacy and Diet Quality in Young Vegans, Lacto-Ovo Vegetarians, Pescatarians, Flexitarians and Omnivores. Public Health Nutr..

[51] Markowski K.L., Roxburgh S. (2019). If I Became a Vegan, My Family and Friends Would Hate Me: Anticipating Vegan Stigma as a Barrier to Plant-Based Diets. Appetite.

[52] Lea E., Worsley A. (2003). Benefits and Barriers to the Consumption of a Vegetarian Diet in Australia. Public Health Nutr..

[53] Riella G., Teper R. (2014). Probabilistic Dominance and Status Quo Bias. Games Econ. Behav..

[54] Alcorta A., Porta A., Tárrega A., Alvarez M.D., Vaquero M.P. (2021). Foods for Plant-Based Diets: Challenges and Innovations. Foods.

[55] Ghaffari M., Rodrigo P.G.K., Ekinci Y., Pino G. (2022). Consumers’ Motivations for Adopting a Vegan Diet: A Mixed-Methods Approach. Int. J. Consum. Stud..

[56] Judge M., Fernando J.W., Begeny C.T. (2022). Dietary Behaviour as a Form of Collective Action: A Social Identity Model of Vegan Activism. Appetite.

[57] Rosenfeld D.L., Burrow A.L. (2017). Vegetarian on Purpose: Understanding the Motivations of Plant-Based Dieters. Appetite.

[58] Malterud K., Siersma V.D., Guassora A.D. (2015). Sample Size in Qualitative Interview Studies: Guided by Information Power. Qual. Health Res..

[59] McHugh P., Smith M., Wright N., Bush S., Pullon S. (2019). If You Don’t Eat Meat… You’ll Die. A Mixed-Method Survey of Health-Professionals’ Beliefs. Nutrients.

[60] Carstensen L., Hartel C. (2006). Chapter 3: Motivation and Behavioral Change. When I’m 64. National Research Council (US) Committee on Aging Frontiers in Social Psychology, Personality, and Adult Developmental Psychology.

