# Supplementary material for: “If I Didn′t Do Sports, I Would Definitely Eat Less Meat”—Physical Activity: Enemy or Ally for Healthier Food Choices?
Source: Nutrients. 2025 Oct 25;17(21):3362. doi: 10.3390/nu17213362 (PMC12609474; doi:10.3390/nu17213362)
Supplement: Supplementary file 1 [file nutrients-17-03362-s001.zip › nutrients-3925517-supplementary.pdf]

File S1 Focus group outline

The focus group was conceived as a semi-structured interview and included the following steps/questions:

1) Standardized introduction to explain the purpose of the focus group and the organization.
2) Ice-breaking opening question: "What is your relationship with food? Do you keep track of nutrients? Do you follow any particular diet?"
3) Open question: "In your opinion, are meat and fish essential for nutrition? What about eggs and cheese?"
4) Open question: "In your opinion, is eating animal protein important for physical activity?"
5) Interactive word cloud – framing QR code – Mentimeter interface: "Imagine starting a totally plant-based diet. What would be the **positive** aspects? Write down as many words as you can think of." The conductor emphasized the importance of answering spontaneously, without overthinking, and then shared the screen. Participants could type words in real time and see a word cloud take shape, along with the responses of all the other participants. When the cloud became stable because no one was writing any more words, the conductor read the most frequent words and asked to elaborate further, starting a deeper discussion.
6) Similar to point 5: Interactive word cloud – framing QR code – Mentimeter interface: "Imagine starting a totally plant-based diet. What would be the **negative** aspects instead? Write down as many words as you can think of." Again, a discussion was opened on the aspects identified by participants as most relevant in a negative way.
7) Open-ended questions: The conductor shared a PPT slide on the screen that summarized the four primary sources of plant proteins: legumes, grains, seeds, and nuts, giving examples of how these sources can be used in everyday dishes. Then, participants were asked whether they consumed these products, how often, and why.
8) Interactive survey – framing QR code – Mentimeter interface: The conductor asked participants to frame a new QR code and answer the following question: "Thinking about plant-based protein dishes, such as the ones we just discussed, how would you rate them?". At that point, the Mentimeter screen was shared with a table that contained eight pairs of adjectives in the form of a semantic differential: bland/tasty, poor/nutritious, unhealthy/healthy, inferior/superior, unsatisfying/fulfilling, difficult/easy, boring/various, exclusive/inclusive. Participants were asked to vote by moving the slider on a 10-point scale. The adjective presented in negative connotation was assigned a score of 1 (e.g., unsatisfactory), while the positive adjective was assigned a score of 10 (satisfactory). After allowing time for all participants to vote, the given scores were observed, paying special attention to the most unbalanced or unusual responses. In this regard, the presenter asked again if, among the people who had given the various responses, there was anyone who wanted to give reasons for their choice.
9) Similar to point 8: Interactive survey – framing QR code – Mentimeter interface: "Thinking about people who follow a fully plant-based diet, how would you rate them?". In this case, the adjectives were: sad/happy, weak/strong, pale/rose, flabby/muscular, and female/ male. Again, the motivations behind the ratings given by the participants were investigated. Adjectives for items 8 and 9 were determined based on previous studies addressing the most common stereotypes about plant-based food and vegan people. These were discussed through an internal briefing among the project researchers.
10) Recap of what was shared throughout the session. Participants were asked if they would like to add any additional and relevant aspects.
11) Finally, participants were debriefed about the session and the project in more detail. They were thanked and provided with all the necessary contact information for the researcher(s) should they have any questions or insights.

Table S1 Main themes emerging from the focus groups and examples of arguments produced by the participants

		**Omn Sed**	**Omn Act**	**Veg*an Sed**	**Veg*an Act**
**RQ1: The role of physical activity in modulating attitudes towards plant-based sources**	Theme I: Comparing the effectiveness of animal and plant proteins for performance	Doubts, importance of being advised by a professional<br>*"In my opinion, it is not necessary to take meat in this case (…) Maybe once in a while, yes, but it doesn't affect, in my opinion, at the level of physical performance in lifting weights or doing even another kind of sport. For example, doing combat sports, where technically you need a lot of animal protein intake, however, there are some cases of athletes who do combat sports without taking animal protein. So, in my opinion, in the end, it is very subjective."*	Animal protein is essential for sports<br>*"Animal protein is more bioavailable than plant protein. I was a vegetarian for two years while I was practicing competitive sport, and during that period I started eating meat again, and my performance totally changed for the better."*	Importance of being advised by a professional<br>*"The only precaution is to have a competent person follow your diet."*	Animal protein is not essential; plant-based protein is more energetic<br>*"I always talk about my experience as an example. When I was training before, I was doing five days a week of calisthenics, plus I was doing trail and Shaolin, … I was never affected, and I was able to put on 4 kg of muscle mass in about three and a half months. So, I would say that even the structure of the fictional noble proteins … is likely scientifically outdated."*
	Theme II: Sources of knowledge	Tradition<br>*We quite follow a Mediterranean diet, so we try to balance a bit of everything during the week, to eat fish, meat, vegetables, and legumes. We try to eat a little bit of everything. I'm not super careful about nutritional values."*	Physicians; coaches<br>*"I had been given information by some physicians years and years ago, and they had said that plant-based protein would not be enough compared to meat, fish, and eggs. I am biased because I love fish and meat and could not do without them."*	Internet; documentaries; social media<br>*"I follow (…) a friend, who is a bodybuilder and personal trainer. He is also an activist; he shows the exercises he does, so definitely, as far as the aspect of putting on muscle mass related to animal food or not, it's a pretty disputable issue."*	Physicians; nutritionists; videos<br>*"…my physician was nice because although they are old generation, when I said that I was vegan, the first thing he told me was: - you will never have a cholesterol problem-."*
	Theme III: Social stigma	Lack of support/services	Exclusivity/social isolation	Feeling of being misunderstood	Fear of becoming a burden

**RQ2: The cons of plant-based choice**		*"I wrote about different family diets because I still live with my parents and they do the grocery shopping; so, if my dad buys steaks and they're about to go bad, what would I do if I had a completely plant-based diet?"*	*"When I think of a time when a large group of people eat, I personally think of a barbecue ... it begins with the preparation of several hands. Considering my personal experience and the fact that I don't have many vegan friends, it would be difficult for me to imagine such a gathering if I followed a plant-based diet."*	*"It's hard to make people around you understand why you made this choice, and they make you look like the crazy one in the family."*	*"I am not strictly vegan because I think it's sometimes hard to be vegan in social settings; so, I also don't want to impose my choices on others or force others to always go along with my choices."*
	Theme IV: Renunciation	Attachment to meat flavor<br>*"I am a die-hard meat fan, so it would be unsatisfying for me to eat only plant-based protein; however, it is a very personal matter."*		Nostalgia for the taste of cheese<br>*"I used to be a big fan of cheese. That was the hardest part when I think back, that's the only part I miss."*	
	Theme V: Logistics and organization	High culinary skills to match meat flavors<br>*"I don't like to cook. Eating totally plant-based for me would mean taking the carrot out of the refrigerator and munching on it. It would really be a problem for me to switch to a plant-based diet because there are very few alternatives without having to go into preparation."*		Difficulties in finding vegan options at restaurants<br>*"It is inconvenient when you are out and about to eat, and there are not always vegetarian or vegan alternatives. In general, there is less choice when you are a vegetarian and even more so when you are vegan."*	
	Theme VI: Inner strength	Difficulty in resisting temptations<br>*"I say strong because... Certainly, times have changed, but in our culinary culture, it is not easy to be vegan; not so much towards the rest of the world, but really towards one's own ... To be able to maintain that willpower for which there is not even the doubt - "I only consume plant-based foods and that's it" - without ever giving in to the temptation or without ever having any interest in doing so, because of ethical intentions or whatever. (…). It's also much effort to follow such a diet from a mental point of view anyway. So always be careful that the alcohol you consume doesn't have animal colorants, that everything you consume doesn't have something*		Tenacity in being an outsider<br>*"In my opinion, people who make a certain choice and go against a large mass of other people who make a different choice in the same context are people who pursue a goal with tenacity and therefore are strong."*	Consciousness and courage<br>*"In my opinion, it is really an aspect related to conscience. I used to consider myself a hypocritical person to love animals and then to find them on my plate. It is really a matter of consciousness, for me also a matter of courage."*

		animal in it. It can be very tiring to keep up with that. (…). You can never be peaceful."			
**RQ2: The pros of plant-based choice**	Theme VII: Ethics	Just mentioned		Strong emotional drive<br>*"I started seeing videos of intensive factory farms and mistreatment, and I couldn't bring myself to close my eyes anymore."*	
	Theme VIII: Health	Healthier, better digestion, gut health<br>*"Digestion of vegetables takes place at the level of the gut primarily, and all vegetables are essential for the bacterial flora of the gut, and on the gut depend immune defenses, etc."*	Lighter, low-fat<br>*"In a plant-based diet, all the things I can think of are prepared with much less seasoning: there is no butter, there are no fats typical of meat and sausages, so just the sensation on the palate when I eat vegetables is of greater lightness."*	The way animals are treated makes meat unhealthy<br>*"Animals are not treated well. You just need to see how they live to understand that it is not good for you to eat them. [...] I saw once that there was pus in the milk; it's not healthy."*	Prevention of cancer and cardiovascular diseases<br>*"I was thinking of cancer and cardiovascular pathologies. So cardiac events, cardiovascular events, obviously strokes, as well as several cancers related to meat ingestion, such as intestinal cancers."*
	Theme IX: Sustainability	Just mentioned		Very knowledgeable<br>*"Eighty percent of the cultivated land is used to feed animals; if that 80 percent of the land were cultivated just for us humans, we would have five times as much food as we have today, so serious problems like hunger could also be solved."*	
